# Endosulfan induces male infertility

**DOI:** 10.1038/cddis.2015.368

**Published:** 2015-12-17

**Authors:** R Sebastian, S C Raghavan

**Affiliations:** 1 Department of Biochemistry, Indian Institute of Science, Bangalore 560 012, India

Pesticides encompass a large group of molecules that are widely used around the globe for improving the efficiency and yield of farming. Many a times their benefits are overshadowed by harmful effects on non-target organisms including humans.^1^ Among the pesticides that have a detrimental effect on human health, organochlorine pesticides top the list, owing to their high transport potential and molecular properties.^2, 3^

Endosulfan (ES) is an organochlorine pesticide associated with harmful side effects on humans that include speculated infertility and teratogenic outcomes.^4^ There are no molecular studies to investigate the underlying mechanism of action of ES in resulting cellular damage, genomic instability and ill health. A recent study by Sebastian and Raghavan^5^ demonstrated the mechanism of ES-mediated testicular toxicity and male infertility using a mice model system.

Comparing the experimentally validated bioavailability in mice and the known concentrations of ES reported in accidental and occupational exposure in humans, we resorted to physiologically relevant sublethal concentrations of ES in the investigation. At the whole-body level, ES induced more weight fluctuations in males as compared with females. RBC, WBC and platelet levels were also affected upon ES exposure, indicating toxicity. CD19^+^ cells too showed variations in treated animals, suggesting impact at the level of the lymphoid system.

Detailed histopathological analyses revealed that among all the organs, liver, lungs and testes were affected, whereas brain, intestine and kidney showed no sign of toxicity. This was also reflected at the functional level.

In testes, high degree of atrophy and tubular necrosis was seen, with many seminiferous tubules having fully or partially depleted spermatogonial mother cells and spermatids. Further detailed analyses through TUNEL assay revealed long-term testicular cell death, indicative of persistent damage.

Following exposure to ES, fluorescence activated cell sorting analysis of different spermatogenic cell populations at various time points spanning spermatogenesis showed remarkable reduction in G1 (spermatocytes and quiescent mother cells), S (dividing mother cells), G2/M (4*n*) and 1*n* (spermatids) cells. These results indicate that ES treatment significantly perturbed a complete cycle of spermatogenesis, causing testicular atrophy and depletion of cell populations.

Although sperms appeared normal with intact head, hook and tail morphology, an immediate reduction in the chromatin integrity was observed upon ES treatment, as confirmed by sperm dispersion assay, which could be attributed to increase in reactive oxygen species (ROS) levels. Interestingly, a dramatic reduction in the sperm count upon ES exposure was observed. While significant reduction in actively motile sperms was noted, an increase in nonmotile sperms was also evident due to testicular cell damage and death. These results revealed that the epididymal sperm number and quality were severely compromised upon ES exposure.

Does the dramatic effect on sperm count and motility affect fertility? It appears to be the case. Extensive experiments on mating using ES-treated male mice revealed that the number of infertile males increased upon exposure to ES. This suggests that the reduced sperm count and motility contributed towards infertility in males. However, we did not find any synergistic effects on fertility levels when both males and females were exposed, indicating that ES-induced infertility could be male specific.

In conclusion, using a variety of approaches, the authors demonstrate that, coupled with pathophysiological changes, ES induced maximal effect on testes causing cell death and depleting testicular cell populations. ES-induced changes in testes were spermatogenesis dependent and led to the reduction in sperm quality and quantity, resulting in male infertility (Figure 1c).

Interestingly, treatment with ES led to ROS-mediated DNA damage and elevated levels of error prone DNA repair leading to genomic instability (Sebastian and Raghavan^5^). This observation in conjunction with the physiological effects exerted by ES gives us new insights into the mechanisms of ES-mediated toxicity.

Several organochlorine pesticides are endocrine disruptors, acting as androgen or oestrogen antagonists. Previous studies indicate that ES may act as an androgen receptor (AR) antagonist.^6, 7^ Besides the conventional AR-abundant organs such as testes and prostate, the lung is also rich in AR expression. By docking studies and bioinformatic analyses, we show that ES indeed can bind to the ligand binding site of AR, with a considerable binding energy as compared with that of dihydrotestosterone, its natural ligand, reinforcing such a hypothesis (Figure 1a). Importantly, we found a reduction in the AR-positive Sertoli cells upon ES treatment, indicative of specific depletion of Sertoli cells (Figure 1b). Therefore, the observed organ specificity of ES action may have a link to AR and the molecular outcomes of this aspect need to be further investigated.

This study is of special interest considering the growing concerns about health hazards of pesticides and lack of in-depth understanding of it.^8, 9, 10, 11^ Besides, the questions addressed here are of global concern due to increasing world trade of farm produce and lack of appropriate quality control measures, pertaining to the usage of pesticides in several countries.^11^ This study could be further extended to other spectra of pesticides with such speculated adverse effects and would be a first step towards better rationalised usage of pesticides for the benefit of mankind.

## Figures and Tables

**Figure 1:**
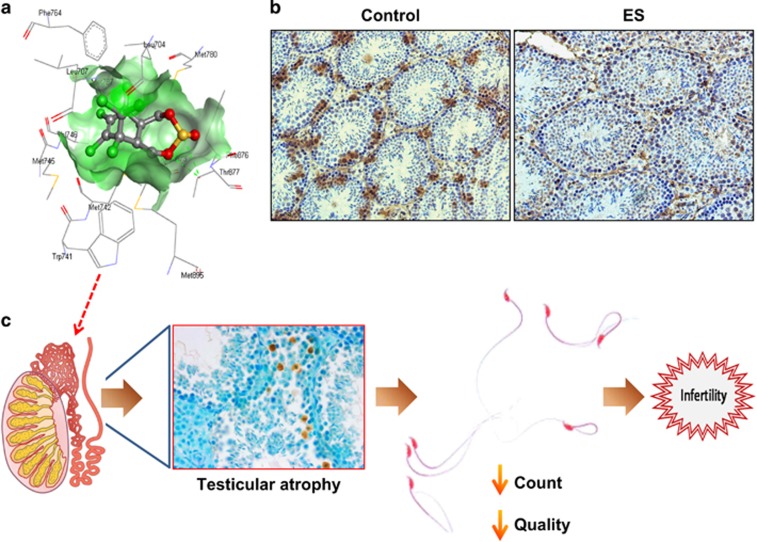
Mechanism of action of ES leading to male infertility. (**a**) Three-dimensional structure of ES binding pattern with androgen receptor using docking studies. Hydrogen bond with Thr877 of androgen receptor (AR), an important bond that facilitates their interaction, is represented in dotted green line. (**b**) Immunohistochemistry showing reduced expression of AR in ES-treated testes sections. (**c**) Events leading to male infertility upon ES exposure. Probable AR antagonism may be responsible for organ specificity of ES action. In testes, it induces testicular atrophy, depleting cell populations in seminiferous tubules and affecting spermatogenesis, which in turn results in qualitative and quantitative reduction in epididymal sperms-culminating in male infertility
